# Colorectal mucinous adenocarcinoma with signet-ring cells: a case report

**DOI:** 10.1186/s12876-026-05055-2

**Published:** 2026-06-26

**Authors:** Yuan-Sheng Liu, Wei-Ting Zeng, Song-Yan Ma, Hui-Qiong Cao

**Affiliations:** 1 Department of Gastroenterology, Huizhou Third People’s Hospital, Guangzhou Medical University, Huizhou, Guangdong China; 2 Department of Pathology, Huizhou Third People’s Hospital, Guangzhou Medical University, Huizhou, Guangdong China

**Keywords:** Mucinous adenocarcinoma, Mucinous adenocarcinoma with signet-ring cells (MAC-SRC), Conventional adenocarcinoma, Colonoscopy, Biopsy, Case report

## Abstract

**Background:**

In recent years, only a few cases of mucinous adenocarcinoma with signet-ring cell carcinoma (MAC-SRC) have been reported, and detailed endoscopic imaging features remain poorly characterized. This is likely because these tumors are often already at an advanced stage at diagnosis, rendering endoscopic examination infeasible. We herein report a young Asian male diagnosed with sigmoid colon MAC-SRC (60% mucinous adenocarcinoma, 40% signet-ring cell carcinoma).

**Case presentation:**

A 27-year-old male presented with recurrent vomiting for 20 days. Abdominal computed tomography showed marked thickening of the sigmoid colon wall. Colonoscopy demonstrated luminal stenosis and rigidity, with extensive erosive lesions covered by abundant, difficult-to-irrigate white mucus; the lesion measured 8 cm in length. Biopsy confirmed mucinous adenocarcinoma. The patient underwent left hemicolectomy, and postoperative pathology verified MAC-SRC. Four months after initiating chemotherapy, the patient continues treatment with no changes in lifestyle, diet, or work compared to the pre-illness state.

**Conclusions:**

Compared with conventional adenocarcinoma, MAC-SRC in young patients shows distinct clinical features and carries a high risk of missed diagnosis. Colonoscopy with adequate biopsy is essential. This patient initially presented with upper gastrointestinal symptoms, leading to a delay in diagnosis. Timely intervention was initiated following endoscopic detection of mucosal abnormalities and confirmatory biopsy findings. The endoscopic characteristics we describe provide diagnostic evidence for MAC-SRC and have important clinical implications.

## Background

Colorectal cancer (CRC) is one of the most common malignant tumors worldwide and the fourth leading cause of cancer-related mortality [1–3]. Conventional adenocarcinoma (AC) represents the most prevalent histological subtype of colorectal malignancy [4, 5]. Colorectal mucinous adenocarcinoma (MAC, accounting for 10–15% of CRC cases) and signet-ring cell carcinoma (SRCC, accounting for 1% of CRC cases) are adenocarcinomas characterized by excessive mucin production and a relatively poor prognosis [6, 7]. Colorectal mucinous adenocarcinoma is defined by abundant extracellular mucin, typically constituting more than 50% of the tumor mass [8]. In contrast, signet-ring cell carcinoma is distinguished by intracellular mucin that displaces the nucleus, with such cells accounting for more than 50% of the tumor [9–11]. In some cases of mucinous adenocarcinoma, intracellular mucin accumulation may lead to signet-ring cell morphology, and these tumors are classified as mucinous adenocarcinoma with signet-ring cells (MAC-SRC) [12]. To date, few case reports of MAC-SRC have been published, and its endoscopic characteristics remain poorly defined. This gap is likely due to the advanced tumor stage at diagnosis, which often makes endoscopic examination infeasible. In this report, we present a case of a young Asian male diagnosed with MAC-SRC in the sigmoid colon, and we evaluate its clinical symptoms, white-light endoscopic findings, CT imaging features, histopathological characteristics, and treatment course.

## Case presentation

A 27-year-old male was admitted to our hospital in January 2026 with a 20-day history of recurrent vomiting. He also reported anorexia, intermittent abdominal pain, 2–3 bowel movements per day, and occasional mucus in stool. Treatment with omeprazole and mosapride yielded no symptomatic improvement. Gastroscopy revealed no abnormalities. He denied fever, dysphagia, constipation, or hematochezia. He had an unintentional weight loss of approximately 10 kg over the previous month. He had a 7-year history of smoking one pack of cigarettes daily and drinking 200 g of alcohol per day. He had no history of diabetes, tuberculosis, or drug allergies, and no family history of cancer. Physical examination revealed mild abdominal tenderness, predominantly in the left lower quadrant, without abdominal guarding or rebound tenderness.

**Fig. 1:**
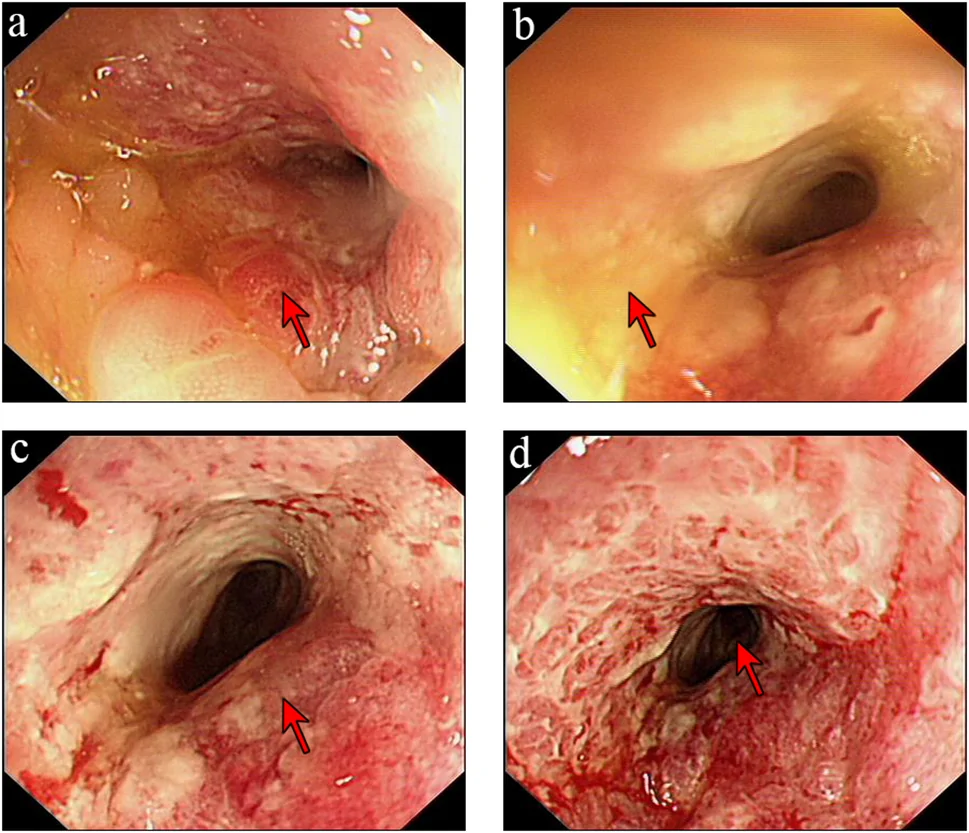
Colonoscopic findings: **a** Mucosal elevation and thickening were noted on initial insertion (red arrow). **b** Copious white mucus was observed after advancing the endoscope (red arrow). **c** After thorough irrigation to clear the mucus, mucosal ulcers, erosions, and friable mucosa with bleeding were evident (red arrow). **d** Severe luminal stenosis was present in the distal sigmoid colon (red arrow)

**Fig. 2:**
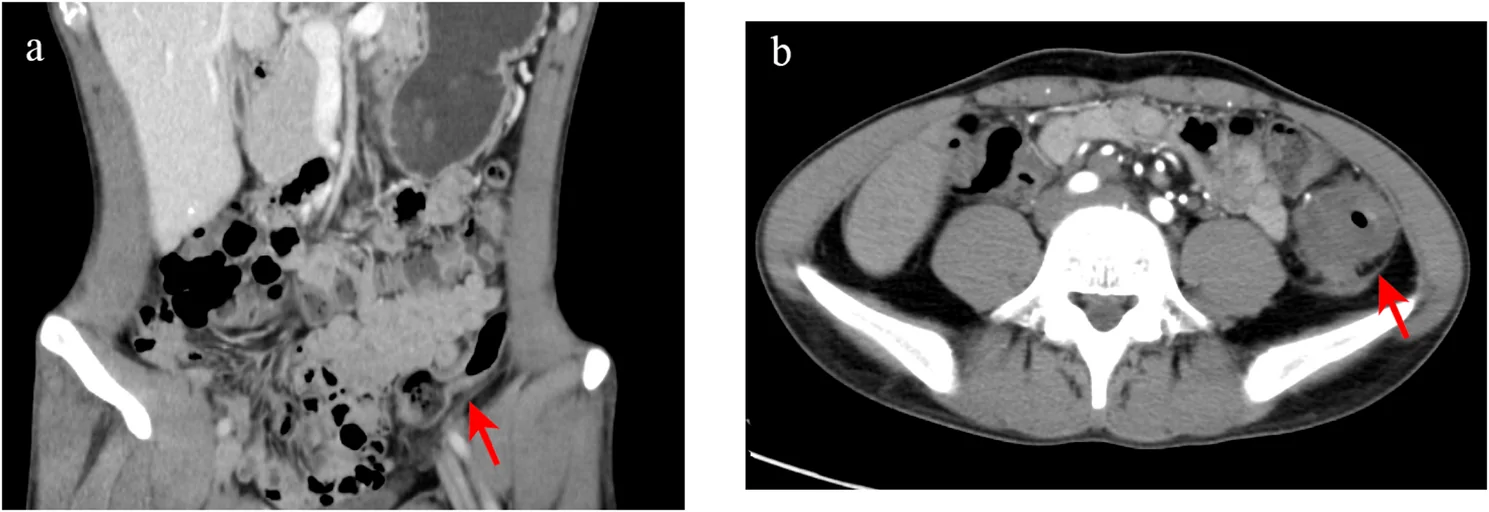
Abdominal computed tomography (CT): **a** Coronal view demonstrates stenosis of the sigmoid colon lumen. **b** Axial view shows marked wall thickening accompanied by several mildly enlarged regional lymph nodes

**Fig. 3:**
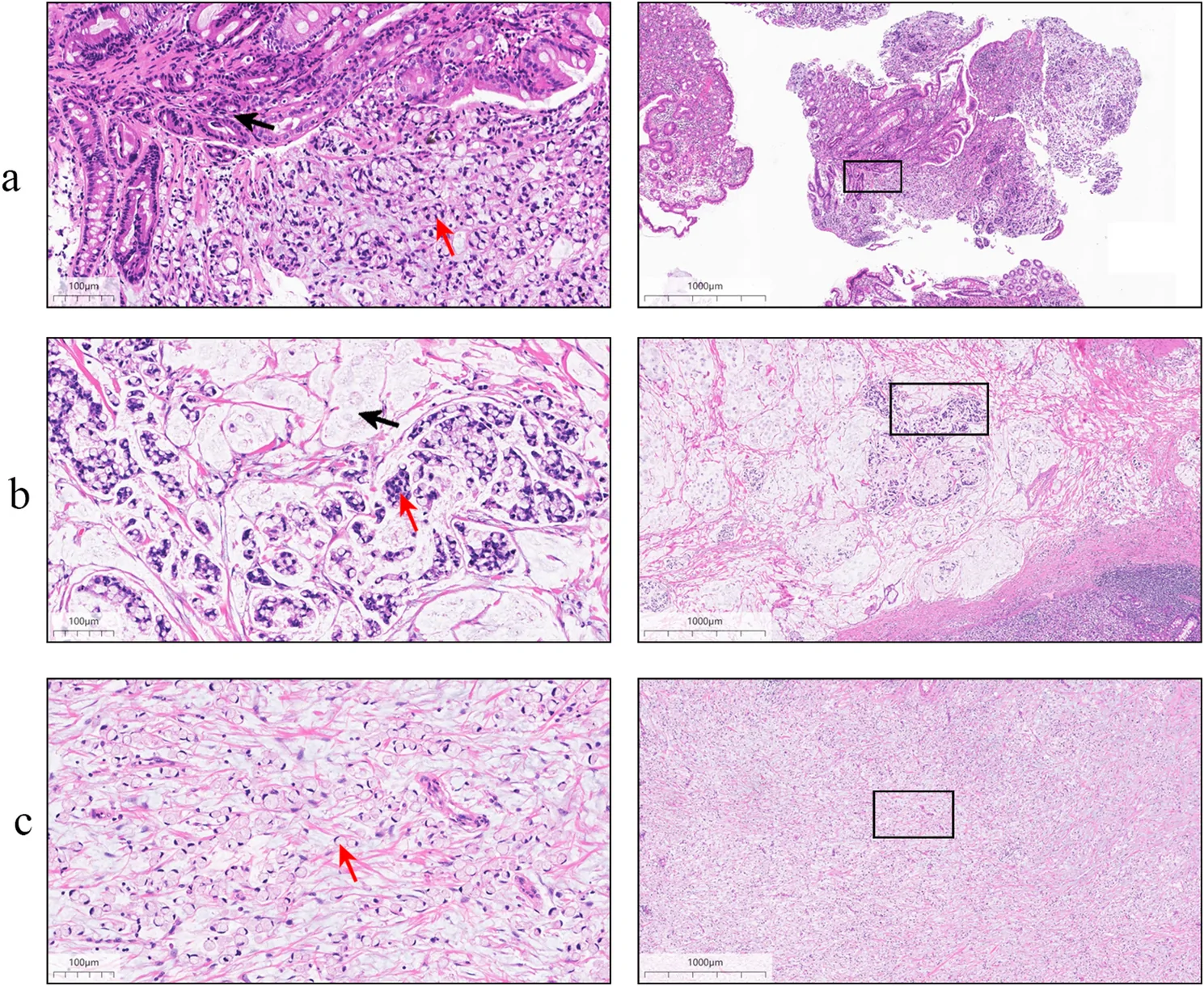
Histopathological features of tumor tissues stained with hematoxylin-eosin (H&E). **a** Colonoscopic biopsy specimens: abundant adenocarcinoma cells infiltrating the mucinous area (red arrow); normal intestinal glands (black arrow) (left, 200× magnification). Overall characteristics of the biopsy tissue (right, 40× magnification). **b** Postoperative pathological samples: abundant adenocarcinoma cells infiltrating the mucinous area (red arrow); mucinous lakes are present in the superficial muscular layer and extracellular matrix (black arrow) (left, 200× magnification). Low magnification is used to assess the proportion of mucinous adenocarcinoma within the entire tumor (right, 40× magnification). **c** In the deep muscular layer of postoperative specimens, mucin deposition is absent, and the lesion is predominantly composed of abundant signet‑ring cells (left, 200× magnification). Low magnification is used to assess the proportion of signet‑ring cell carcinoma within the entire tumor (right, 40× magnification)

Laboratory tests showed a white blood cell count of 10.8 × 10⁹/L, a neutrophil count of 10.05 × 10⁹/L (93.4%), and a hemoglobin level of 136 g/L. Liver function, renal function, electrolyte levels, coagulation parameters, and infectious disease serologic markers were within normal limits. Colonoscopy (SonoScape EC-P560) revealed stenosis of the sigmoid colon approximately 28 cm from the anal verge, with rough, fine-granular mucosa, intestinal rigidity, and wall thickening (Fig. 1a). Further advancement showed a large amount of white mucus that was difficult to rinse (Fig. 1b). After thorough irrigation, mucosal ulcers, erosions, and easy mucosal bleeding were observed (Fig. 1c). Distal advancement identified severe luminal stenosis that could not be traversed by a standard endoscope (Fig. 1d). An ultra-thin endoscope (SonoScape EG-P550N) was then used to pass the stenotic segment, and no abnormalities were detected in the remaining intestinal mucosa. The total length of the lesion was approximately 8 cm. We performed a deep biopsy to obtain specimens at multiple sites. To further clarify the diagnosis, we performed relevant examinations. Tumor markers, C-reactive protein, and erythrocyte sedimentation rate were within normal ranges. A T-cell spot test for tuberculosis was positive. However, the patient had no sputum; therefore, no specimen for acid-fast staining was collected, and chest CT revealed no abnormalities. No additional evidence suggested intestinal tuberculosis. Contrast-enhanced abdominal CT demonstrated significant thickening of the sigmoid colon wall with several mildly enlarged regional lymph nodes (Fig. 2). Multiple biopsies confirmed mucinous adenocarcinoma (Fig. 3a).

The patient was transferred to the Department of General Surgery and underwent left hemicolectomy. Postoperative pathology confirmed adenocarcinoma with a mucinous adenocarcinoma component (approximately 60%) and a signet-ring cell carcinoma component (approximately 40%) (Fig. 3b, c). The two tumor types were distinguished at 200× magnification, and their respective proportions within the entire tumor were determined at 40× magnification. The cancer infiltrated the entire bowel wall and penetrated the visceral peritoneum, with vascular and perineural invasion. Metastatic carcinoma was found in 9 of 13 lymph nodes and in the lateral peritoneal tissue. Pathological stage: mixed-type adenocarcinoma of the colon, pT4a, N2b, M1c.

The patient subsequently received chemotherapy with the following regimen: bevacizumab 7.5 mg/kg, oxaliplatin 130 mg/m², and capecitabine 1000 mg/m² orally on days 1–14 every 3 weeks. Two months after the start of chemotherapy, the patient was in good general condition, with occasional abdominal distension and a weight gain of 2.5 kg. At the four-month follow-up, the patient reported having completed the sixth cycle of chemotherapy, with two additional cycles remaining. During chemotherapy, adverse reactions including poor appetite and nausea occurred but resolved completely after a four-day rest period. A repeat abdominal CT scan revealed mild postoperative edema of the intestinal wall. Thereafter, the patient’s work, daily activities, and dietary habits returned to normal. Regular follow-up was performed.

## Discussion and conclusions

Epidemiological data show regional variations in the incidence of colorectal mucinous adenocarcinoma (MAC). The incidence in Asian countries is 3.9%, while in Western countries it ranges from 10% to 13.6% [13–15]. Clinically, MAC occurs more frequently in the proximal colon than in the rectum or distal colon [16, 17]. Compared with conventional adenocarcinoma (AC), MAC affects a higher proportion of female and younger patients and is more often diagnosed at an advanced stage [18, 19]. The incidence of MAC-SRC is approximately 2% in Western countries and may be lower in Asia [12]. The case reported here involves a 27-year-old Asian male with no family history of cancer, representing a rare clinical scenario. His clinical manifestations included recurrent vomiting, anorexia, intermittent abdominal pain, and occasional mucoid stools, which differ from the classic symptoms of colorectal cancer. This highlights the importance of recognizing atypical tumor manifestations in young patients and the necessity of gastrointestinal endoscopy or abdominal CT for evaluation. Preoperative evaluation of colorectal cancer mainly relies on CT. On imaging, MAC shows more prominent eccentric intestinal wall thickening than conventional adenocarcinoma. After contrast enhancement, MAC demonstrates poor enhancement of solid components and heterogeneous tumor enhancement [20]. Our patient had no signs of intestinal obstruction or contraindications to colonoscopy; thus, colonoscopy and biopsy were performed to confirm the diagnosis.

Small-sample studies have shown that excessive mucin secretion in MAC tumors leads to a lower diagnostic yield of endoscopic biopsy for MAC than for conventional adenocarcinoma [21]. We used a deep-tissue biopsy technique: after an initial superficial sample, closed forceps were reinserted into the lesion and opened to obtain deeper tissue, confirming MAC histopathologically. Endoscopically, MAC-SRC presents with stenosis, ulceration, and copious white mucus resistant to irrigation. Its endoscopic features are different from those of colorectal serrated adenoma. The colorectal serrated adenoma exhibits two distinct types: polypoid, characterized by exophytic growth with infolded fibromuscular stroma and a lobulated or cauliflower-like appearance in advanced stages, and superficial (flat), which is slightly elevated or flat, never exceeds twice the thickness of adjacent normal colorectal mucosa, and lacks such stroma [22, 23]. The endoscopic appearance of MAC-SRC may be attributed to its mucinous adenocarcinoma cells and signet-ring cells. Mucinous adenocarcinoma secretes copious mucus, producing macroscopically visible white mucous lakes [24]. In contrast, signet-ring cells typically exist as single cells or loose clusters and infiltrate diffusely throughout the intestinal wall [25]. Owing to the biological behavior of signet-ring cells, MAC-SRC is more aggressive than mucinous adenocarcinoma without a signet-ring component and tends to present with intestinal stenosis at an earlier stage [12, 26]. Colonoscopic findings of mucosal mucus and stenosis may be misdiagnosed as Crohn’s disease, intestinal tuberculosis, or colonic diverticulitis. However, relevant examinations did not provide sufficient diagnostic evidence; consequently, the final diagnosis of mucinous adenocarcinoma was established based on pathological findings. Therefore, biopsy is critical and our method may improve the diagnostic yield of tumor biopsy.

There are significant differences in metastatic patterns between MAC and AC. AC commonly metastasizes to distant organs such as the liver, whereas MAC is more prone to peritoneal metastasis and MAC has a higher postoperative lymph node positivity rate than AC [27, 28]. Our pathological results also confirmed peritoneal rather than liver metastasis in this MAC-SRC patient, which was consistent with the patient’s upper gastrointestinal symptoms. The mechanism underlying this metastatic difference is thought to be closely related to the mucin component [29]. The mucin component may increase intestinal epithelial permeability, allowing mucin to enter the peritoneal cavity and regional lymph nodes, promoting peritoneal and lymph node metastasis [30, 31]. Early lymph node and peritoneal metastasis were also observed in this case.

Compared with AC, MAC has a very poor prognosis. The 5-year overall survival rate of patients with colorectal MAC is significantly lower than that of patients with AC at the same site [32]. Reports indicate that MAC-SRC patients have an even worse prognosis than MAC patients, and SRC is considered an independent predictor of poor prognosis [33, 34]. A previous analysis showed that the 5-year overall survival rate of colorectal MAC-SRC patients was 42.5%, between that of MAC and SRC patients [35]. The presence of signet-ring cells in mucin pools and high mucin content predict aggressive clinical behavior in colorectal MAC, which explains the high invasiveness and metastatic potential of these tumors [25, 36]. The patient in this case remains under regular follow-up.

In conclusion, we report a case of a 27-year-old male with colorectal MAC-SRC who presented with upper gastrointestinal symptoms and was at high risk of misdiagnosis. Its endoscopic features are distinctly different from those of conventional adenocarcinoma, characterized by stenosis, ulceration, and copious mucin secretion, which can be easily confused with Crohn’s disease, diverticulitis, and intestinal tuberculosis. Adequate biopsy is essential for confirming the diagnosis. This case emphasizes the need for heightened clinical awareness and proactive evaluation of atypical symptoms in young patients to improve early diagnosis.

## Limitation

Due to the limited follow‑up period, the current assessments reflect only the treatment efficacy observed to date; continued follow‑up is planned.

## Data Availability

No datasets were generated or analysed during the current study.

## Abbreviations

MAC: Mucinous adenocarcinoma
MAC-SRC: Mucinous adenocarcinoma with signet-ring cells
SRCC: Signet-ring cell carcinoma
AC: Adenocarcinoma
CRC: Colorectal cancer
CT: Computed tomography
